# Methylglyoxal interaction with superoxide dismutase 1

**DOI:** 10.1016/j.redox.2019.101421

**Published:** 2020-01-07

**Authors:** Panagis Polykretis, Enrico Luchinat, Francesca Boscaro, Lucia Banci

**Affiliations:** a Interuniversity Consortium for Magnetic Resonance of Metallo Proteins (CIRMMP), via Luigi Sacconi 6, 50019, Sesto Fiorentino, Florence, Italy; b Magnetic Resonance Center - CERM, University of Florence, via Luigi Sacconi 6, 50019, Sesto Fiorentino, Florence, Italy; c Department of Experimental and Clinical Biomedical Sciences “Mario Serio”, University of Florence, viale Morgagni 50, 50134, Florence, Italy; d Mass Spectrometry Center (CISM), University of Florence, via U. Schiff 6, 50019, Sesto Fiorentino, Florence, Italy; e Department of Chemistry, University of Florence, via della Lastruccia 3, 50019, Sesto Fiorentino, Florence, Italy

**Keywords:** Methylglyoxal, Superoxide dismutase 1, Glycation, AGEs, Oxidative stress

## Abstract

Methylglyoxal (MG) is a highly reactive aldehyde spontaneously formed in human cells mainly as a by-product of glycolysis. Such endogenous metabolite reacts with proteins, nucleotides and lipids forming advanced glycation end-products (AGEs). MG binds to arginine, lysine and cysteine residues of proteins causing the formation of stable adducts that can interfere with protein function. Among the proteins affected by glycation, MG has been found to react with superoxide dismutase 1 (SOD1), a fundamental anti-oxidant enzyme that is abundantly expressed in neurons. Considering the high neuronal susceptibility to MG-induced oxidative stress, we sought to investigate by mass spectrometry and NMR spectroscopy which are the structural modifications induced on SOD1 by the reaction with MG. We show that MG reacts preferentially with the disulfide-reduced, demetallated form of SOD1, gradually causing its unfolding, and to a lesser extent, with the intermediate state of maturation – the reduced, zinc-bound homodimer – causing its gradual monomerization. These results suggest that MG could impair the correct maturation of SOD1 *in vivo*, thus both increasing cellular oxidative stress and promoting the cytotoxic misfolding and aggregation process of SOD1.

## Highlights

• MG forms stable adducts with the immature forms of SOD1.
• MG causes the unfolding of the apo-SOD1^SH^ form.
• MG causes the monomerization of the E,Zn-SOD1^SH^ form.
• In both forms, arginine 143 is more prone to interact with MG.
• The structural modifications caused by MG impair the correct maturation of SOD1.

## Graphical abstract

**Figure:**
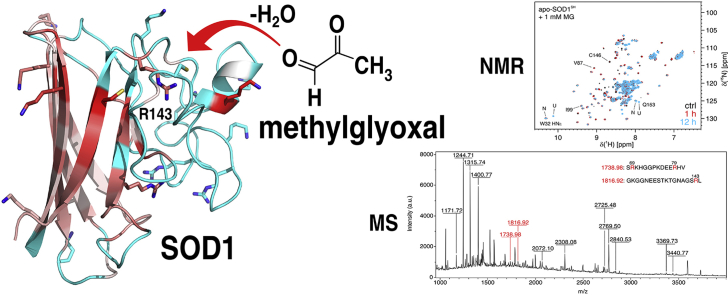

## 1 Introduction

While the human body comes in contact with most toxic substances through the diet, through the environment, or generally through exposure to exogenous agents, some endogenous compounds, which are derived from the physiological cell metabolism, can be extremely harmful, if the mechanisms dedicated to their neutralization fail. This is the case of methylglyoxal (MG), a highly reactive α-oxoaldehyde, potentially formed in all mammalian cells mainly as a by-product of the metabolism of the triosephosphate intermediates of glucose [1,2]. Specifically, during glycolysis, MG is formed from the spontaneous degradation of glyceraldehyde-3-phosphate (GAP) and dihydroxyacetone phosphate (DHAP) [[3], [4], [5]]. Literature reports that about 0.1%–0.4% of the glycolytic flux results in the formation of this highly reactive dicarbonyl [6]. Additional precursors of MG include amino-acetone, derived from the catabolism of proteins [1,2,7], and ketone bodies, derived from lipid peroxidation [8].

The detoxification of MG and other reactive aldehydes is performed by the glyoxalase system [9]. In eukaryotes, such system is composed by two cytosolic thiol-dependent catalytic enzymes: during a first step, glyoxalase I (Glo1) catalyses the isomerisation of the hemithioacetal, formed by the spontaneous reaction of MG with glutathione (GSH), to the intermediate S-d-lactoylglutathione; subsequently, glyoxalase II (Glo2) converts S-d-lactoylglutathione to d-lactate, restoring the GSH consumed during the first reaction [9].

**Fig. 1:**
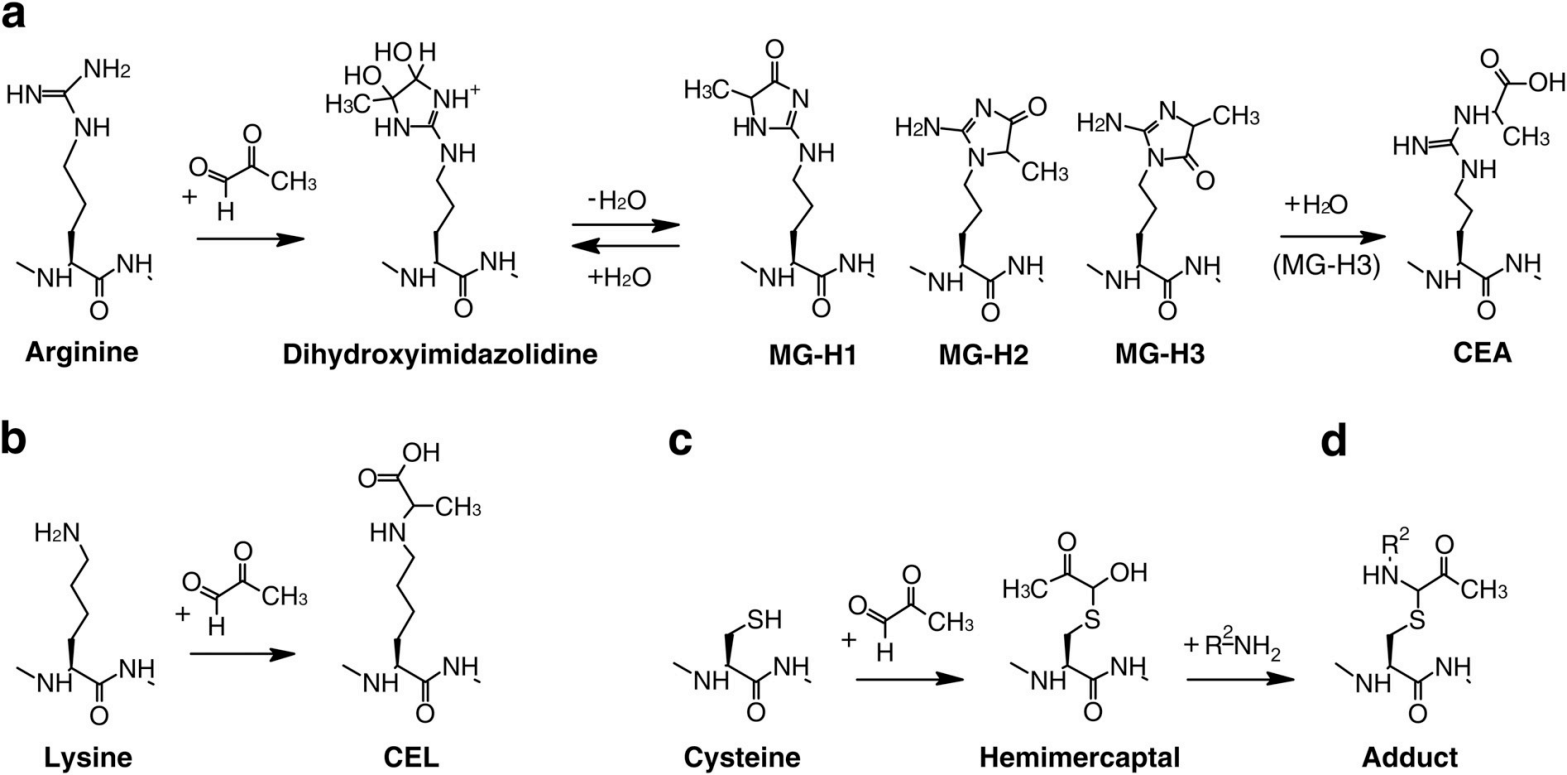
MG reaction with amino acid residues of proteins. MG reacts with arginine forming MG-H1, MG-H2 and MG-H3 isoforms, the last of which generates CEA upon hydrolysis (**a**); MG reacts with lysine forming CEL (**b**); MG reacts with cysteine forming hemimercaptal (**c**), which in turn can further react with a free amino (NH_2_) group forming a stable adduct (d).

Intracellular MG can reach micromolar concentrations [10], and its levels reflect a balance between the cellular glycolytic rate and the detoxification efficiency of the glyoxalase system [3]. Specifically, the tissue levels of MG have been quantified in mice, in which it can reach concentrations up to 4.3 μM in the hepatocytes [11]. MG is a potent electrophile that reacts with proteins, nucleotides and phospholipids forming stable adducts called advanced glycation end-products (AGEs) [5]. MG is known to irreversibly modify proteins by spontaneously reacting with the side chains of arginine, lysine and cysteine residues [[12], [13], [14], [15]]. Reaction of MG with the guanidinium group of arginine leads to the formation of three possible isomers of methylglyoxal hydroimidazolone (MG-H1, MG-H2 and MG-H3); subsequently, MG-H3 can form N^7^-carboxyethyl arginine (CEA) upon the addition of a water molecule (Fig. 1a) [15]. When MG interacts with the ε-NH_2_ of lysine, it forms N-ε-(carboxyethyl)lysine (CEL, Fig. 1b). MG can also interact with the sulfhydryl group of cysteine forming a hemimercaptal (Fig. 1c) [13]. Moreover, CEL and the cysteine hemimercaptal can potentially form inter or intra-molecular adducts by reacting with a NH_2_ group from the same or other molecules (Fig. 1d) [13].

The reaction between MG and proteins, nucleotides or lipids, which results in the gradual accumulation of AGEs in tissues, has been related with aging and with several pathologies including hypertension, cardiovascular diseases, diabetes, cancer and neurodegenerative diseases [[3], [13], [16], [17], [18], [19], [20]].

Due to their high rate of glucose consumption and oxidative metabolism, neurons are likely more exposed to MG than other types of cells [3]. In fact, several studies demonstrated the neurotoxic effects of MG associated with oxidative stress and the accumulation of AGEs [[3], [21], [22], [23], [24], [25], [26]].

Among the affected proteins, it has been reported that MG can react with the metalloenzyme superoxide dismutase 1 (SOD1), causing the loss of enzymatic activity due to the release of the copper ion from Cu,Zn-SOD1 catalytic site [27], together with the formation of MG-induced protein aggregates [28]. Similarly, it has been shown that glyoxal, another highly reactive dialdehyde able to form AGEs, can react with SOD1 to form stable adducts [29]. Furthermore, *ex vivo* experiments reported that SOD1 extracted from erythrocytes of diabetic patients is significantly more glycated and has a lower enzymatic activity, with respect to controls [30]. SOD1 is an essential anti-oxidant metalloenzyme that catalyses the dismutation of O_2_^•-^ to H_2_O_2_ and O_2_ with very high reaction rates [[31], [32], [33]]. Consistent with a decrease of intracellular SOD1 activity, it has been shown that MG causes a significant increase of intracellular NO and O_2_^•–^ levels [34]. Furthermore, it has been hypothesised that the release of the highly reactive copper ion in the cellular environment could contribute to increase the production of reactive oxygen species (ROS) [26]. Therefore, the MG-induced inactivation of SOD1 has been related to the increase of oxidative stress, that in turn is associated with aging and other pathological states [18]. Despite these premises, it is not yet clear whether MG-induced decrease of SOD1 cellular activity occurs as a consequence of MG reacting with the fully mature, disulfide-containing enzyme, Cu,Zn-SOD1^SS^, or instead the reaction between MG and the immature forms of SOD1 is responsible for the incomplete protein maturation and subsequent structural destabilization, that could lead to the loss of enzymatic activity reported in the literature.

Therefore, we sought to investigate the reaction between MG and the immature forms of SOD1 through Nuclear Magnetic Resonance (NMR) spectroscopy and Mass Spectrometry (MS), to determine whether MG has preferential reactivity for one of these forms, and which structural modifications occur on the protein. Specifically, we focused on the initial state of the protein after synthesis: the apo, disulfide-reduced monomer (apo-SOD1^SH^), which is partially unfolded, exposes the dimer interface residues to the solvent and has a higher aggregation propensity [35], and on the zinc-containing, disulfide-reduced dimer (E,Zn-SOD1^SH^), which is a stable intermediate maturation state that precedes chaperone-assisted copper binding and disulfide bond formation [[36], [37], [38]].

## 2 Materials and methods

### 2.1 Protein purification and demetallation

The human recombinant SOD1 was purified implementing an existing protocol [39]. *E. coli* BL21(DE3) Gold cells were transformed with a pET28a plasmid encoding the wt SOD1 gene. The cells were grown at 37 °C in LB medium (or in ^15^N-labelled M9 medium for the NMR experiments), supplemented with 100 μM ZnSO_4_, until mid-log phase. Consequently, the cells were induced with 0.5 mM isopropyl-β-D-1-tiogalattopiranoside (IPTG), and grown for an additional 4 h at 30 °C. The cells were then harvested, re-suspended in 20 mM Tris, 50 μM ZnSO_4_, 1 M DTT, pH 8, supplemented with protease inhibitor tablets (cOmplete ULTRA, EDTA-free, Roche) and lysed by sonication (3 s ON, 10 s OFF, at 60% of amplitude for 40 min). After clarification, the lysate was loaded on a DEAE Sepharose Fast Flow (GE Healthcare) anion exchange column and eluted with NaCl gradient. The fractions containing SOD1 were further purified by a Superdex 75 26/600 column (GE Healthcare) size exclusion column, eluting with 20 mM Tris, 150 mM NaCl, pH 8. The fractions containing SOD1 were collected and checked by SDS-PAGE (AnyKD, Bio-Rad).

hSOD1 was demetallated as previously described [[40], [41]]. Briefly, the protein underwent 10 repeated dialyses (at least 8 h each): 7x dialysis against 10 mM EDTA in 50 mM acetic acid at pH 3.7, followed by 1x dialysis against 50 mM acetic acid at pH 3.7, and 2x consecutive dialyses against 50 mM acetic acid at pH 5.5. Finally, the buffer was replaced with PBS, pH 7.4. The reduction of the disulfide bond of SOD1 was performed by incubating the apo-SOD1^SS^ with 50 mM of DTT for 40’ at 37 °C. Finally, DTT was removed washing the buffer with oxygen-free PBS under anaerobic conditions.

### 2.2 Incubation of SOD1 with MG and SDS-PAGE

Two 60 μM samples of unlabelled apo-SOD1^SH^ and E,Zn-SOD1^SH^ (the metallation state was previously checked by 1D ^1^H NMR), in oxygen-free PBS buffer, were divided in 500 μL aliquots. A solution of 1 M of MG (Sigma-Aldrich) was prepared dissolving the α-oxoaldehyde in PBS and adjusting the pH to 7.1. Consequently, the protein samples have been exposed to 0 (control), 1, 5 and 30 mM of MG for a period of 5, 24 and 48 h, at room temperature and under anaerobic conditions. Such range of concentrations has been chosen to be comparable with that used in the previous studies [[27], [28]]. At the aforementioned time intervals, a protein fraction was taken from each sample, it was diluted to 10 μM, and was subjected to a denaturing and reducing SDS-PAGE using a 3x FSB (150 mM Tris-HCl, 300 mM DTT, 6% SDS, 0.3% bromophenol blue, 30% glycerol, pH 6.8) solution.

With the aim to perform MS experiments, a further incubation of the protein with MG was performed using two 90 μM samples of unlabelled apo-SOD1^SH^ and E,Zn-SOD1^SH^, in oxygen-free PBS buffer. The protein samples were divided again in 500 μL aliquots, 5 mM of MG solution were added to each one and were incubated at 37 °C, under anaerobic conditions. At the time intervals of 1, 5, 24 and 48 h, 10 μL of protein solution from each sample were subjected to a reducing and denaturing SDS-PAGE, and the remaining protein samples, were analysed by MALDI-TOF, after replacing the buffer with 0.02% acetic acid pH 3.8 (suitable for MS measurements), in order to remove the MG and stop the reaction.

### 2.3 MS experiments

The aforementioned apo-SOD1^SH^ and E,Zn-SOD1^SH^ samples in acetic acid 0.02% pH 3.8 were concentrated in the range of 40–60 μM and were analysed MALDI-TOF on a Bruker Daltonics Ultraflex III MALDI TOF/TOF instrument. 1 μL of protein sample was mixed with a 1 μL of matrix solution (SA 10 mg/mL in 70:30 [v/v] acetonitrile : water with 0.1% TFA). Comapass for flexSeries 1.4 was used as data acquisition software in positive linear mode. The instrument had been previously calibrated using the Bruker Protein I calibration standard kit (5000–17000 Da).

The apo-SOD1^SH^ and E,Zn-SOD1^SH^ samples incubated with 5 mM of MG for 1 h, at 37 °C, have been selected for a further MALDI-TOF analysis upon digestion with chymotrypsin. The abovementioned protein samples, along with the corresponding controls, were subjected to SDS-PAGE (15 μM of protein per well) and the resulting bands were excised and transferred in 1.5 mL microcentrifuge tubes. Each band underwent three consecutive washes in 40 μL of acetonitrile for 10 min, followed by a final wash in 40 μL of 0.1 M ammonium bicarbonate (AMBIC). The solution was removed and the gel bands were digested with chymotrypsin (Promega, Madison, WI). Briefly, 10 μL of a 100 ng/μL of the chymotrypsin dissolved in 25 mM AMBIC supplemented with 10 mM CaCl_2_ were added to the gel bands. After overnight digestion at 37 °C, the supernatant was recovered and the reaction was stopped adding 1 μL of 10% TFA. For the MALDI-TOF analysis, 0.5 μL of matrix solution (α-cyano-4-hydroxycinnamic acid (HCCA) 20 mg/mL in 70:30 [v/v] acetonitrile : water with 0.1% TFA) were mixed with 0.5 μL of the digested protein fragments on an AnchorChip™ target (600 μm Ø). The instrument was calibrated using the Bruker Peptide calibration standard kit (1046–3147 Da) and afterwards the spectra were acquired in positive ion reflectron positive mode over the *m*/*z* range 900–4000.

### 2.4 NMR experiments

Time-resolved NMR experiments were performed at a 950 MHz Bruker AVANCE III HD spectrometer equipped with a 5 mm TCI triple-resonance cryoprobe. Samples of [U–^15^N]-apoSOD1^SH^ (50 μM) and [U–^15^N]-E,Zn-SOD1^SH^ (100 μM) in oxygen-free PBS buffer, pH 7.4, 10% D_2_O were first analysed in the absence of MG and subsequently mixed with MG (1 mM final concentration). The reactions were carried out at 37 °C in the NMR spectrometer and monitored by alternating 1D ^1^H spectra (zgesgp, 2 min 17 s each) and 2D ^1^H, ^15^N SOFAST-HMQC spectra [42] (sfhmqcf3gpph, 20 min 19 s each). The NMR spectra were processed with Bruker Topspin and subsequently analysed with Bruker Dynamics Center to retrieve signal intensities as a function of time and to perform non-linear curve fitting with a mono-exponential function.

## 3 Results

### 3.1 Reaction of SOD1 with MG by SDS-PAGE

**Fig. 2:**
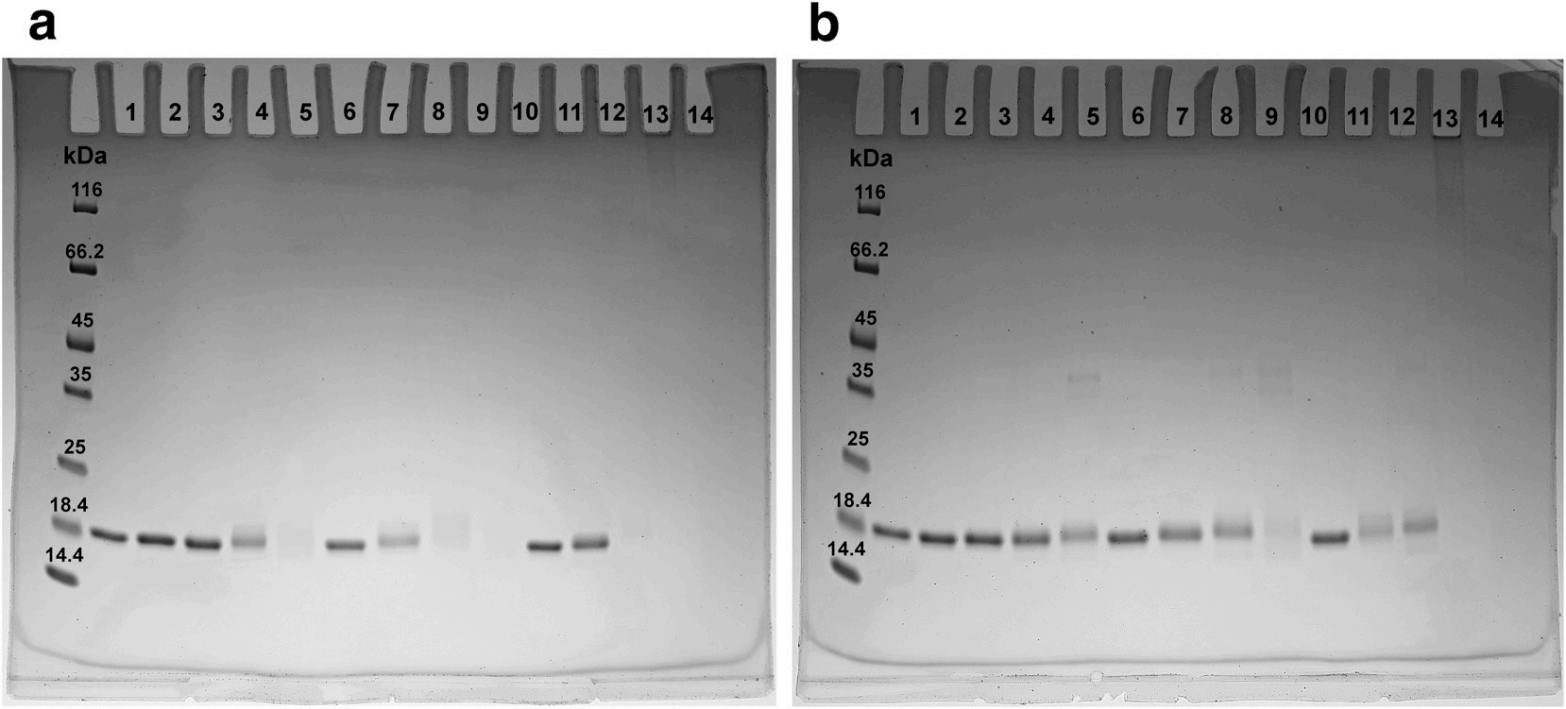
SDS-PAGE electrophoresis performed on the apo-SOD1^SH^ (**a**) and E,Zn-SOD1^SH^ (**b**) incubated with MG. **Lane 1**: control; **lane 2**: no MG, 5 h; **lane 3**: 1 mM MG, 5 h; **lane 4**: 5 mM MG, 5 h; **lane 5**: 30 mM MG, 5 h; **lane 6**: no MG, 24 h; **lane 7**: 1 mM MG, 24 h; **lane 8**: 5 mM MG, 24 h; **lane 9**: 30 mM MG, 24 h; **lane 10**: no MG, 48 h; **lane 11**: 1 mM MG, 48 h; **lane 12**: 5 mM MG, 48 h; **lane 13**: 30 mM MG, 48 h.

**Fig. 3:**
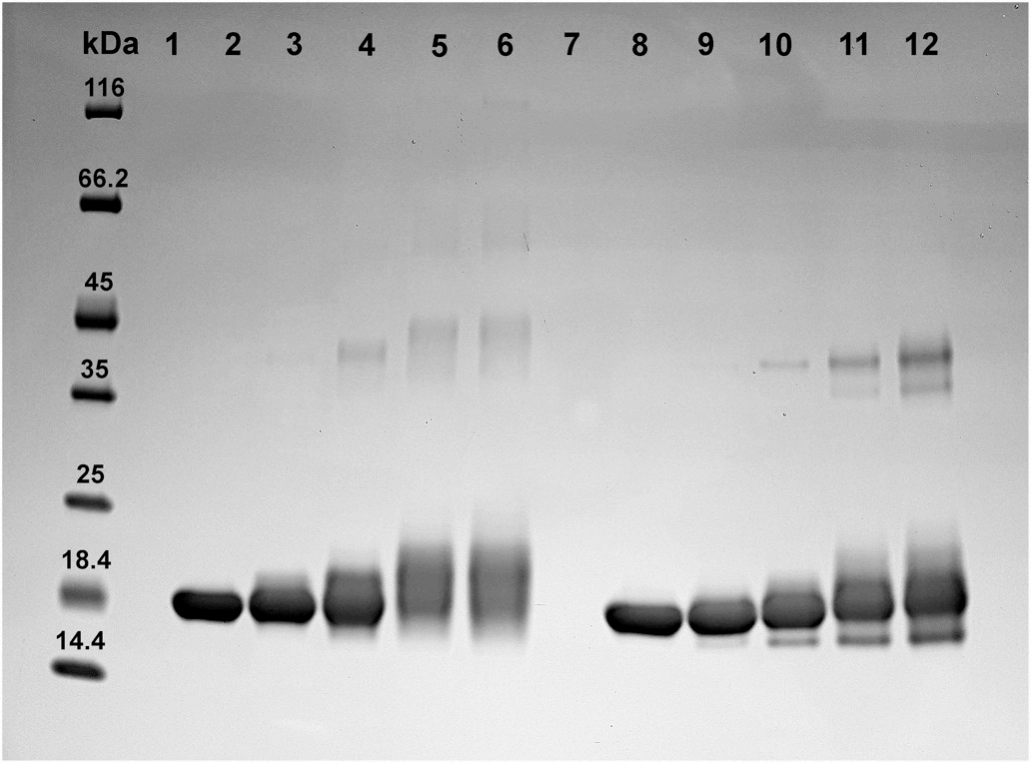
SDS-PAGE electrophoresis performed on the apo-SOD1^SH^ (**left**) and E,Zn-SOD1^SH^**(right**) incubated with 5 mM of MG. Lane **2 & 8**: control; lane **3 & 9**: 1 h; lane **4 & 10**: 5 h; lane **5 & 11**: 24 h; lane **6 & 12**: 48 h.

Gel electrophoresis, performed under denaturing and reducing conditions, clearly indicated that MG reacts with SOD1 forming covalent adducts. Indeed, the exposure of the protein to MG causes an evident band shift, which is time- and dose-dependent (Fig. 2, Fig. 3). Such observations are in accordance with those previously described on Cu,Zn-SOD1 [[27], [28]]. In addition, a somewhat different reactivity is observed between MG and the two forms of the protein. More precisely, SDS-PAGE suggested that MG reacts on numerous sites of apo-SOD1^SH^, forming a multitude of products with different electrophoretic mobility and causing the loss of a defined protein band at high concentrations of MG and/or long incubation times (Fig. 2, Fig. 3), whereas the reaction of MG with E,Zn-SOD1^SH^ seems to react with less sites, producing a better-defined protein band that migrates at a higher molecular weight compared to the unreacted protein (Fig. 2, Fig. 3). Furthermore, the effects of MG at longer incubation times and higher concentrations are much more dramatic on apo-SOD1^SH^, eventually leading to the loss of any detectable protein band, while in the case of E,Zn-SOD1^SH^, reaction with MG under the same conditions led to the formation of the oligomeric forms as those previously observed, which are likely covalent adducts [[27], [28]].

### 3.2 MALDI-TOF analysis identified MG reaction sites

**Fig. 4:**
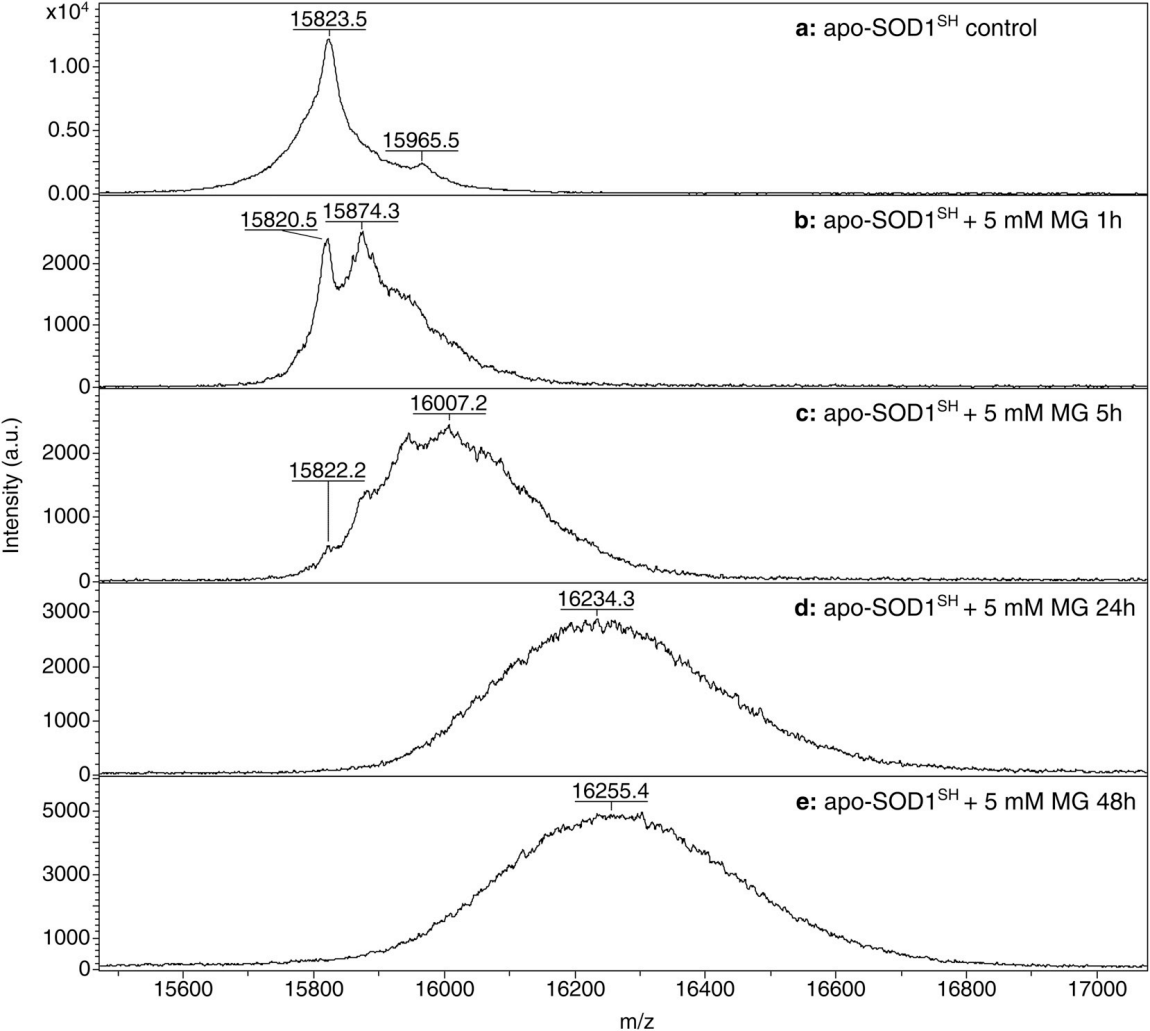
MALDI-TOF spectra of control apo-SOD1^SH^ (**a**), and incubated at 37 °C with 5 mM of MG for 1 h (**b**), 5 h (**c**), 24 h (**d**), and 48 h (**e**).

**Fig. 5:**
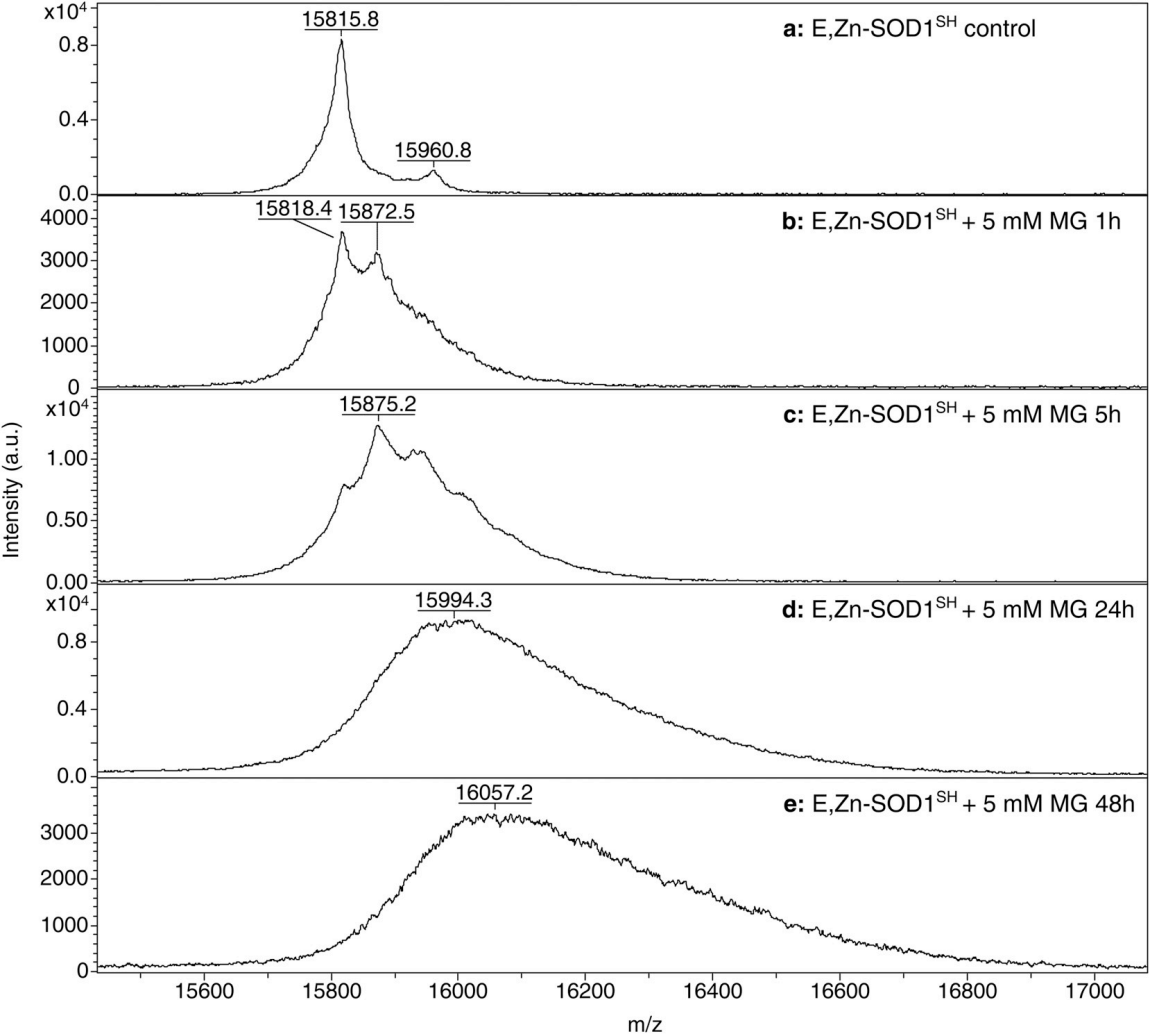
MALDI-TOF spectra of control E,Zn-SOD1^SH^ (**a**), and incubated at 37 °C with 5 mM of MG for 1 h (**b**), 5 h (**c**), 24 h (**d**), and 48 h (**e**).

The effects of MG on SOD1 were also analysed by mass spectrometry (MALDI-TOF), which resulted in a net gain of molecular weight of SOD1 relative to the unreacted protein. In accordance, the molecular ions of unreacted apo-SOD1^SH^ and E,Zn-SOD1^SH^ produced major peaks at *m/z* ratios of 15823.5 and 15815.8, respectively (Fig. 4, Fig. 5a). The obtained masses are consistent, within the instrumental error, with a theoretical molecular mass of human SOD1 of 15823.4 Da, calculated considering the cleavage of the N-terminal methionine, the complete loss of metal ions under MALDI-TOF conditions and the estimated protein charge at pH 3.8. A minor peak at 15965.5 and 15960.8, respectively, was detected in both samples, possibly belonging to a small fraction of protein which underwent an unidentified chemical modification. Protein samples incubated with 5 mM of MG for increasing amounts of time presented remarkably different mass profiles. In fact, in both samples, a marked broadening of the main peak was observed, indicative of the time-dependent formation of multiple MG-SOD1 adducts (Fig. 4b–e and Fig. 5b–e). In accordance with SDS-PAGE, the MALDI-TOF data indicated that the apo form of the protein is more affected by the exposure to MG, as suggested by the peak broadening and shifting to higher *m/z* ratios over time, which is more pronounced in the case of apo-SOD1^SH^, due to the formation of several forms with increasing molecular weights (Fig. 4b–e and Fig. 5b–e).

In a higher molecular mass range, in all MALDI-TOF spectra a minor peak at around 31600–32500 was observed, likely corresponding to a covalent dimer of SOD1, whose level slightly increased over the reaction time (Figs. S1 and S2).

In the sample of apo-SOD1^SH^ incubated with 5 mM of MG for 1 h, we observed the presence of a major peak at 15874.7, adjacent to the peak at 15820.5 that corresponds to the unreacted protein (Fig. 4b). Similarly, in the spectrum of the E,Zn-SOD1^SH^ there is a distinct peak at 15872.5, next to the peak at 15818.4 (Fig. 5b). The presence of these peaks indicated that in both cases, during the first hour of incubation SOD1 reacts preferentially with one MG molecule, which adds 54 Da to the molecular mass of the protein. Such net gain of molecular weight is consistent with the formation of methylglyoxal hydroimidazolone (MG-H1, MG-H2, MG-H3) on the arginine residues [42,43]. After 1 h of incubation with MG, the reacted protein was the predominant species in the case of the apo-SOD1^SH^ (Fig. 4b), while in the case of E,Zn-SOD1^SH^, the unreacted protein was still the predominant species (Fig. 5b), as further evidence that the apo form has a higher propensity to react with MG. However, in both cases, the number of MG-protein adducts increased over time, producing species with different molecular weight and causing the broadening of the peaks due to spectral overlap.

**Fig. 6:**
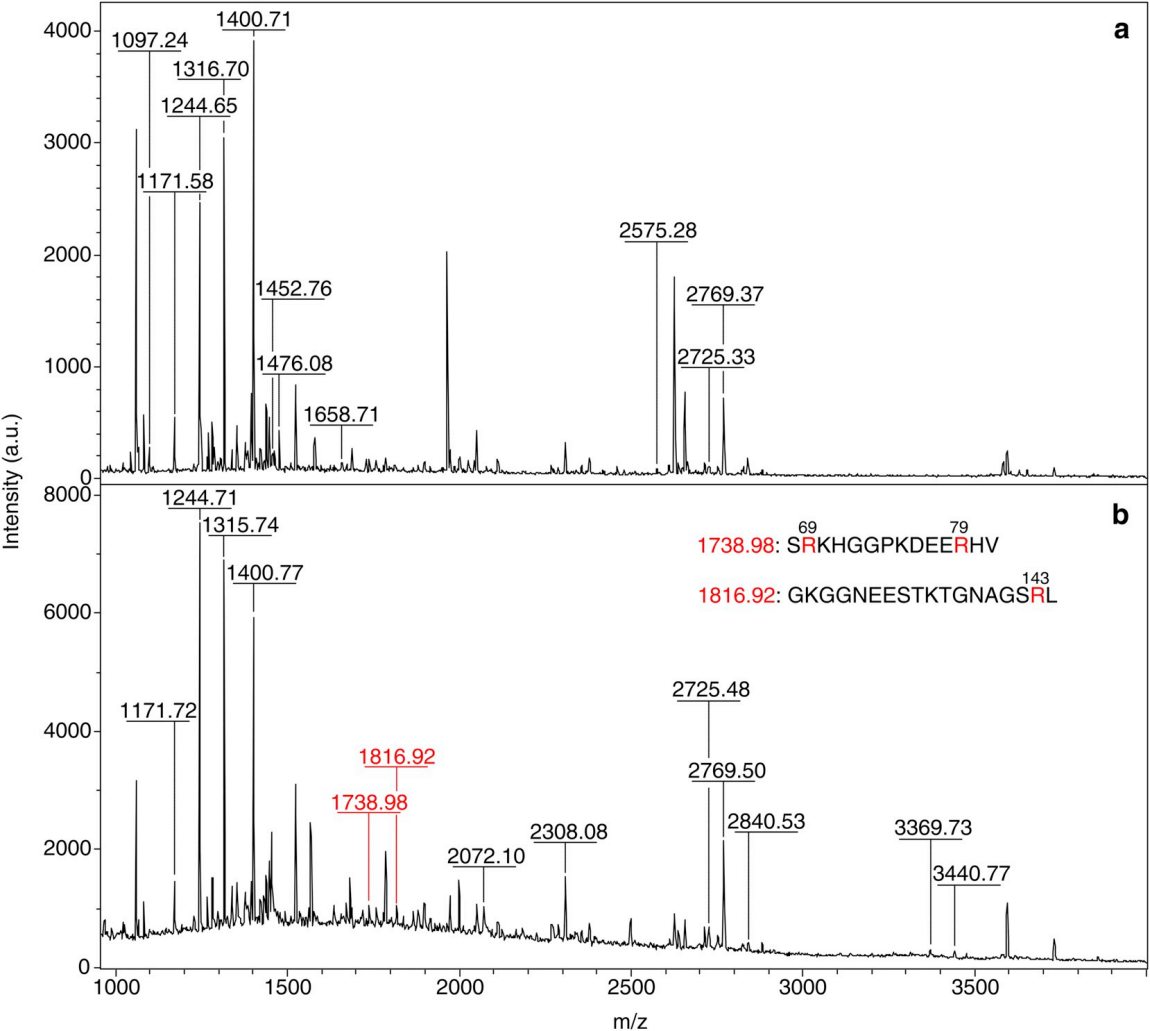
MALDI-TOF spectra of the chymotryptic digests of control apo-SOD1^SH^ (**a**) and apo-SOD1^SH^ incubated with 5 mM MG at 37 °C for 1 h (**b**).

**Fig. 7:**
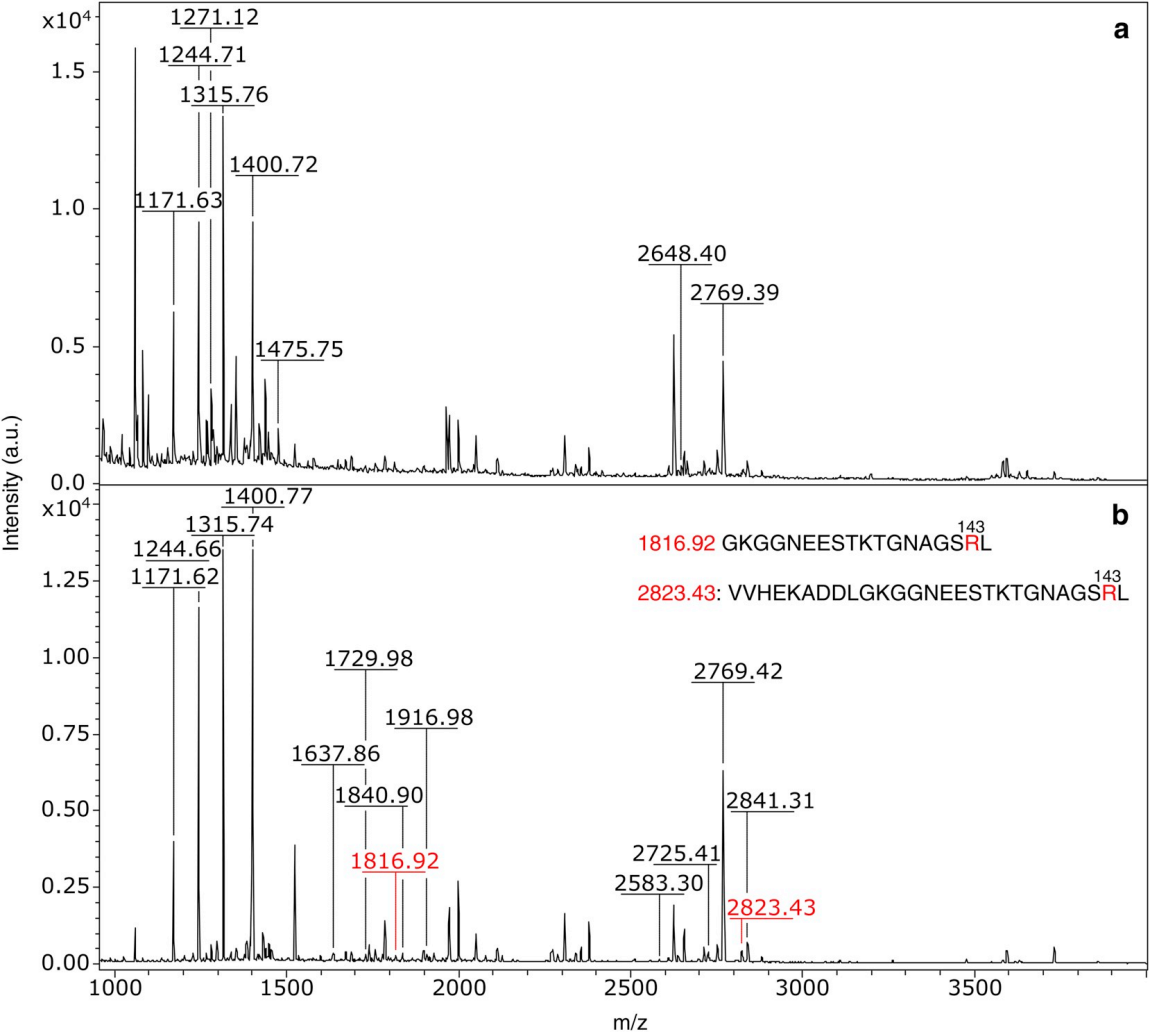
MALDI-TOF spectra of the chymotryptic digests of control E,Zn-SOD1^SH^ (**a**) and E,Zn-SOD1^SH^ incubated with 5 mM MG at 37 °C for 1 h (**b**).

In order to identify which amino acids reacted more likely with MG within the first hour, the corresponding protein samples were further analysed by digestion with chymotrypsin, followed by fragment analysis by MALDI-TOF. The MALDI-TOF analysis performed on the chymotryptic digests of apo-SOD1^SH^ revealed that during the first hour of incubation MG reacted with either R69, R79, or R143 (Fig. 6 and S3). The same analysis performed on E,Zn-SOD1^SH^ revealed the presence of MG adducts with R143 only (Fig. 7 and S4).

In addition to the reaction with arginine which adds 54 Da to the protein mass, literature reported that MG can form a dihydroxyimidazolidine intermediate (Fig. 1) with arginine, which causes a 72 Da increment [44]. Furthermore, a 72 Da increment can derive from the reaction of MG with lysines and cysteines, which are minor MG-derived adducts [[44], [45]]. Therefore, we further investigated the presence of digested fragments compatible with such modifications. This analysis indicated that a 72 Da increment could derive from several modified arginine, lysine and cysteine residues on both apo-SOD1^SH^ (Fig. S3) and E,Zn-SOD1^SH^ (Fig. S4). However, +72 adducts are less abundant in the sample with respect to +54 adducts, as indicated by MALDI-TOF analysis on the intact protein samples after 1 h of reaction.

### 3.3 Time-resolved NMR reveals protein structural changes

**Fig. 8:**
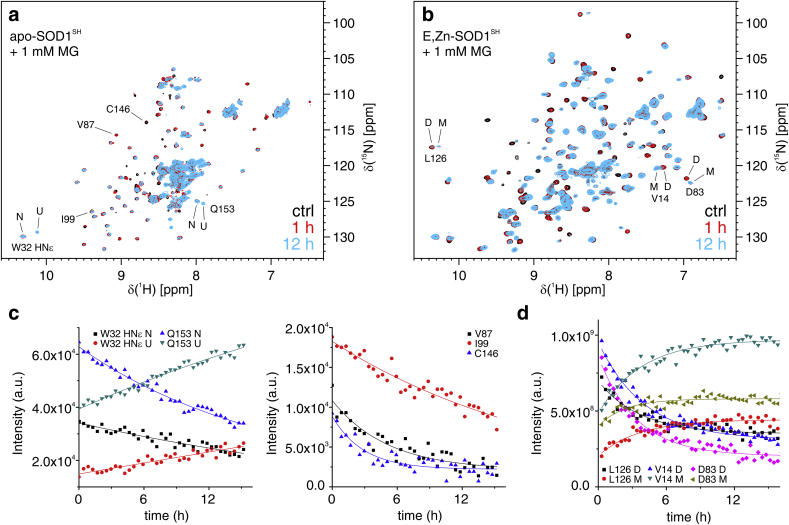
(**a,b**) Overlay of the 2D ^1^H, ^15^N NMR spectra of apo-SOD1^SH^ (**a**) and E,Zn-SOD1^SH^ (**b**) in the absence of MG (black), and during the reaction with 1 MG after 1 h (red) and 12 h (light blue). Representative residues in different states are labelled (N = native, U = unfolded, D = dimer, M = monomer). (**c,d**) Representative plots of signal intensity *vs.* time of the residues of apo-SOD1^SH^ (**c**) and E,Zn-SOD1^SH^ (**d**) labelled in **a** and **b**, respectively, during the reaction with 1 mM MG. Best-fit mono-exponential curves are shown as lines of the corresponding colour. (For interpretation of the references to colour in this figure legend, the reader is referred to the Web version of this article.)

**Fig. 9:**
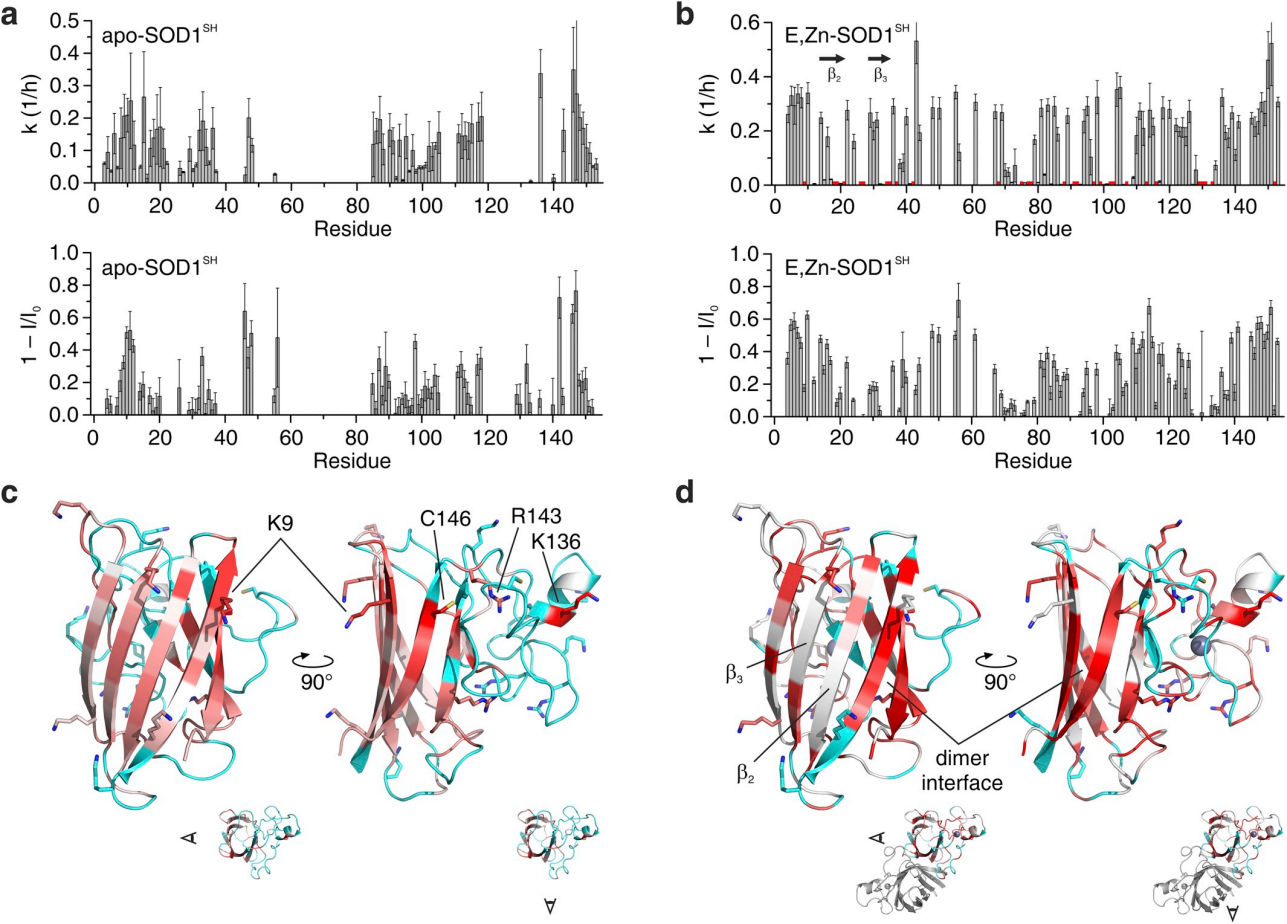
(**a,b**) Plots of apparent rate constants (top) and signal loss at 1 h (bottom) vs. residue number obtained from the reaction of apo-SOD1^SH^ (**a**) and E,Zn-SOD1^SH^ (**b**) with 1 mM MG. Apparent rate constants were obtained from pseudo-first order mono-exponential fittings. In **b**, red dots indicate residues with k = 0. (**c,d**) Cartoon representation of the 3D structure of a SOD1 monomer (PDB 2AF2), colour-coded according to the apparent rate constants of apo-SOD1^SH^ (**c**) and E,Zn-SOD1^SH^ (**d**) from white (k = 0) to dark red (k > 0.4 h^-1^). Unassigned or non-fitted residues are shown in cyan. The viewpoints with respect to the monomer (apo-SOD1^SH^) or the homodimer (E,Zn-SOD1^SH^) are shown at the bottom. Residues close to red spots are labelled in **c**; the homodimer interface and β-strands 2 and 3 are labelled in **d**. (For interpretation of the references to colour in this figure legend, the reader is referred to the Web version of this article.)

In order to obtain structural insights on the reaction between MG and the immature forms of SOD1, we performed time-resolved ^1^H–^15^N 2D NMR experiments on samples of apo-SOD1^SH^ and E,Zn-SOD1^SH^ incubated with 1 mM MG (Fig. 8a and b). The 2D NMR spectra provide information at single-residue resolution, which can be retrieved by signal intensity changes or by chemical shift perturbation. The above data were recorded in a time-resolved fashion during the reaction (Fig. S5), and subsequently fitted with a pseudo-first order kinetic model (Fig. 8c and d). In both experiments, spectral changes were observed over the course of the reaction. In the case of apo-SOD1^SH^, we observed a decrease of intensity of most signals arising from the residues located in the β-barrel, which is the structured region of the apo protein. Simultaneously, a set of signals centred around 8.3 ppm ^1^H increased in intensity (Fig. 8a). This region is typical of unfolded polypeptides, showing that the reaction with MG caused the gradual unfolding of apo-SOD1^SH^. Notably, signals from different residues in the folded protein decreased with different rates (Fig. 8b). This observation can be explained with the fact that reaction of MG on a given position in the structure causes a local perturbation that affects the chemical shifts of the neighbouring residues, but not those of residues far from the reaction site. Hence, reactions at different positions affect different sets of signals in the spectra, and the rate of signal disappearance for each residue is dependent on the number of reaction sites in its proximity. Indeed, decrease rates plotted as a function of residue number revealed that some regions of the protein are more affected by MG reaction (Fig. 9a). Signal loss at 1-h reaction was also plotted to account for fast-disappearing signals that could not be properly fitted. Mapping the perturbed regions on the 3D structure revealed potential reaction sites at close to residues K9, K136, R143 and C146 (Fig. 9c), some of which are consistent with the MALDI-TOF analysis of the chymotrypsin digest (Fig. S3).

Interestingly, a different behaviour was observed in the time-resolved NMR experiment on E,Zn-SOD1^SH^, which is a well-folded homodimer. In this case, only a subset of signals decreased over time, whereas several other signals remained unperturbed. (Fig. 8b). Moreover, the rates of disappearance of the decreasing signals were more uniform (Fig. 9b). This suggested a common cause for the signal loss of residues located at different positions across the protein structure. Simultaneously, a second set of signals in the NMR spectra increased in intensity at a similar rate (Fig. 8d). Notably, these signals are also present in the spectra of unreacted E,Zn-SOD1^SH^, and arise from a small fraction of protein in the monomeric state, which is at the equilibrium with the homodimer [46]. Consistently, the non-decreasing signals arise from residues for which neither monomerization nor reaction with MG cause a change in chemical shift, so that their intensity is proportional to the sum of the two species. The observed pattern of signals therefore may still provide information on the location of MG reaction sites, but also reflects the fact that reaction with MG increases the population of monomeric E,Zn-SOD1^SH^. Mapping the perturbations on the 3D structure confirmed that, indeed, the majority of residues perturbed by the reaction with MG are located in the β-barrel at the homodimer interface, whereas β-strands 2 and 3, which are not at the interface, are not affected (Fig. 9d). Possible reaction sites, such as K136 and C146, are also identified. These results are also compatible with the MALDI-TOF data concerning the reaction rate of MG with R143, which was identified in both samples as a likely reaction site. Indeed, signals arising from residues close to R143 decrease with a higher rate in the apo-SOD1^SH^ NMR spectra with respect to those in the E,Zn-SOD1^SH^ spectra (Fig. S6), suggesting that R143 reacts faster in the demetallated sample.

## 4 Discussion

The data obtained in this study indicated that MG has dramatic effects on the structure of SOD1. Such effects exacerbate with time and with increasing concentration of MG. The long-term effects are difficult to be defined and characterized in detail, because they affect the whole protein structure indistinctly. Thus, we primarily sought to identify the first reactions that cause structural destabilization of the protein and that constitute the first steps of a process that leads to the complete unfolding of the apo-SOD1^SH^, and to the monomerization of E,Zn-SOD1^SH^.

The SDS-PAGE experiments indicated that MG reacts with the apo-SOD1^SH^ and E,Zn-SOD1^SH^ forming stable adducts with different electrophoretic mobility with respect to the control samples. In the case of the apo form such effect exacerbates over time and MG concentration, causing the complete loss of a defined protein band. In accordance with previous studies [[27], [28]], we observed the formation of higher molecular weight oligomers, resistant to reducing and denaturing conditions, confirming that MG acts even as a cross-linking agent. Such crosslinking ability could represent a risk factor for the formation of aggregates, which especially in the case of apo-SOD1^SH^, are considered to be related to the onset of the familial amyotrophic lateral sclerosis (fALS) [[35], [47], [48]]. However, the results of a recent study suggested that glycation inhibits the amyloid aggregation of SOD1 in both, the metallated and the amyloidogenic apo form of the protein [49].

The MALDI-TOF analysis performed on both forms of the undigested protein is in line with that from SDS-PAGE, indicating that MG is able to bind at multiple sites within the protein structure, causing the broadening and the shift of the peaks towards higher molecular weights. During the first hour of incubation under anaerobic conditions, we observed that MG mainly forms one adduct with both forms of SOD1, adding 54 Da to the protein mass. This net weight gain is compatible with the reaction between MG and arginine, which results in the formation of hydroimidazolone (MG-H1, MG-H2, MG-H3), previously identified on other proteins [[43], [44]]. The MALDI-TOF analysis performed on the chymotryptic digests, indicated that in apo-SOD1^SH^ the residues which are prone to react with MG are R69-79-143, while in E,Zn-SOD1^SH^ only R143 reacts with MG. Overall, the metallated form of SOD1 has shown to be more resistant to the glycating effects of MG, and this is likely due to its quaternary conformation which is more structured, exposing fewer residues to the solvent.

Through NMR we monitored at single residue-level the time course of the reaction between MG and the protein. Such analysis has shown that the reaction between the monomeric apo-SOD1^SH^ and MG occurs at multiple sites and gradually leads to the unfolding of the protein. This finding is consistent with a previous report, in which however only the oxidized, i.e. disulfide containing protein, was analysed [49]. Furthermore, NMR experiments demonstrated that exposure to MG causes monomerization of E,Zn-SOD1^SH^. It is noteworthy that, upon interaction with MG, in the spectra acquired on apo-SOD1^SH^, signals arising from the unreacted protein gradually disappear but no new signals are observed arising from the reacted protein. This is likely due to the formation of a multitude of adducts, in which the glycation occurred on different residues, each causing a different conformational rearrangement and therefore a different set of chemical shifts. In such situation the concentration of each adduct remains below the detection limit, thus preventing detection by NMR.

Based on these results, we can propose a mechanism for MG binding effects, where the structure of SOD1 in both states is destabilized upon the formation of an initial adduct, most likely upon binding to R143. This causes a partial loss of the protein tertiary structure and exposes further residues to the solvent and to the reaction with MG. In this way a chain of reactions is likely established, which leads to the formation of a high number of glycations, involving lysine and cysteine residues as well.

SOD1 is an essential component of the cellular anti-oxidant defence system. In parallel, a growing number of studies demonstrated the high susceptibility of neurons to MG toxicity, which acts simultaneously as a promoting factor for both the formation of AGEs and the increment of the intracellular oxidative stress [[3], [21], [22], [24], [25], [26]]. The observed unfolding of apo-SOD1^SH^, and the monomerization of E,Zn-SOD1^SH^, both constitute conformational modifications that prevent the correct maturation of Cu,Zn-SOD1, i.e. the catalytically active form of the enzyme.

Consequently, the structural modifications caused by MG on the immature states of SOD1 described in this study may contribute to the loss of the enzymatic activity of Cu,Zn-SOD1 previously observed *in vitro* [[27], [28]], and *ex vivo* [30].

The function of proteins is strictly dependent on their conformation. Thus, the reaction with a molecule like MG, which is able to form stable adducts with proteins, perturbing their structural conformation, can impair their correct function and catalytic activity. The results of this study describe the effects of MG on the structure of SOD1, and therefore contribute to the understanding of the mechanism by which MG compromises its enzymatic activity, strengthening the link between the glycating action of MG and the increase of intracellular oxidative stress.

## Author contributions

P.P., E.L. and L.B. conceived the work; P.P. and E.L. designed the experiments; P.P. produced the protein samples and performed the reaction with MG; E.L. performed the NMR experiments and analysed the data; F.B. performed the MALDI-TOF experiments; F.B. and P.P. analysed the MALDI-TOF data; P.P., E.L., F.B. and L.B. wrote the manuscript.

## Declaration of competing interest

The authors declare that they have no conflict of interests.
