# Supplementary material for: Methylglyoxal interaction with superoxide dismutase 1
Source: Redox Biol. 2020 Jan 7;30:101421. doi: 10.1016/j.redox.2019.101421 (PMC6957824; doi:10.1016/j.redox.2019.101421)
Supplement: Multimedia component 2 [file mmc2.docx]

Supplementary Information

**Methylglyoxal interaction with superoxide dismutase 1**

**Polykretis, P. ^a^, Luchinat, E.^b,c^, Boscaro F.^d^ & Banci, L.^b,e*^**

^a^Interuniversity Consortium for Magnetic Resonance of Metallo Proteins (CIRMMP), via Luigi Sacconi 6, 50019, Sesto Fiorentino, Florence, Italy.

^b^Magnetic Resonance Center - CERM, University of Florence, via Luigi Sacconi 6, 50019, Sesto Fiorentino, Florence, Italy.

^c^Department of Experimental and Clinical Biomedical Sciences “Mario Serio”, University of Florence, viale Morgagni 50, 50134, Florence, Italy.

^d^Mass Spectrometry Center (CISM), University of Florence, via U. Schiff 6, 50019, Sesto Fiorentino, Florence, Italy.

^e^Department of Chemistry, University of Florence, via della Lastruccia 3, 50019, Sesto Fiorentino, Florence, Italy.

^*^To whom correspondence should be addressed. Telephone: +39 055 457 4273. E-mail: banci@cerm.unifi.it

Supplementary Figures S1-S5


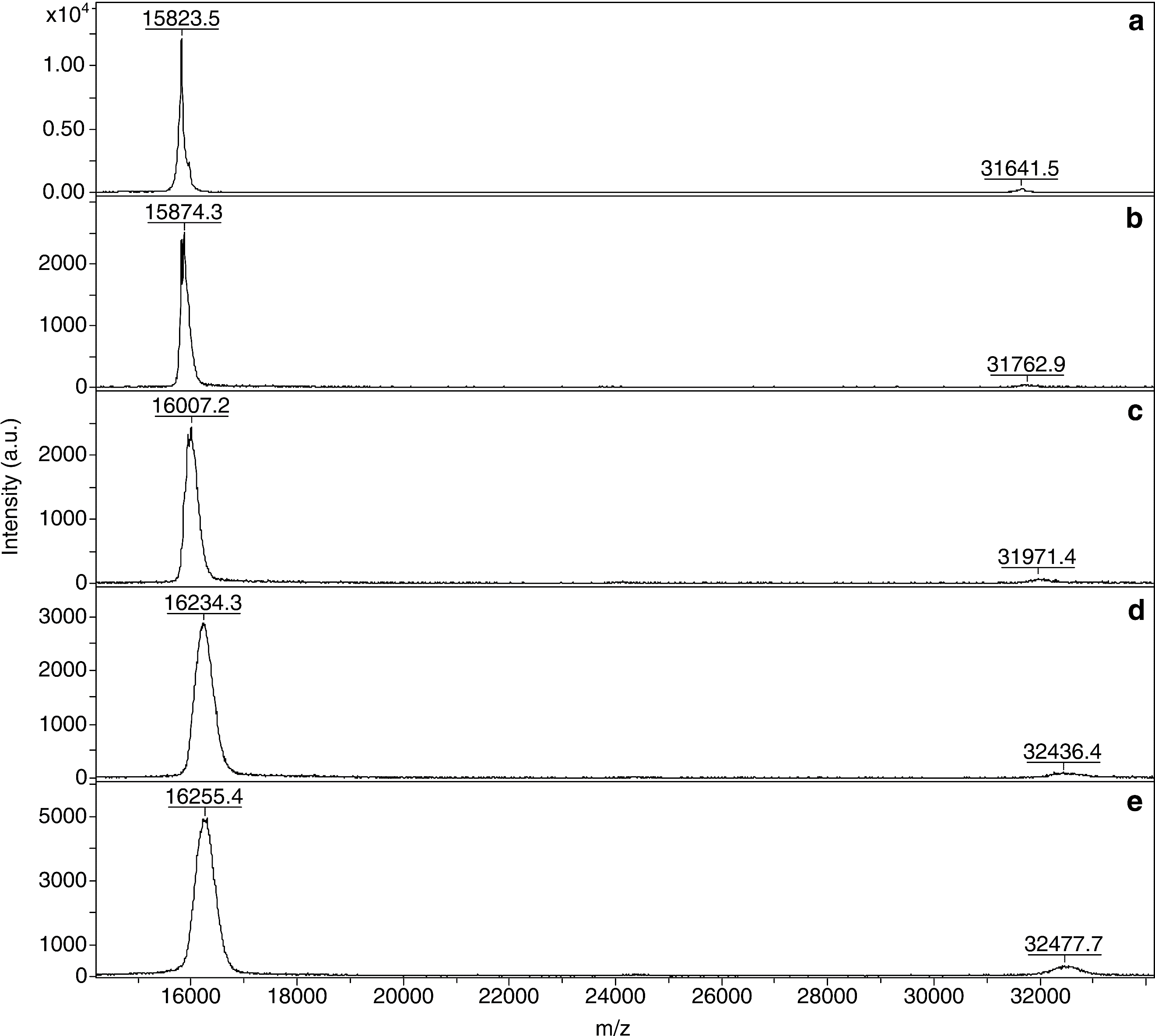


**Figure S1** High *m/z* range MALDI-TOF spectra of control apo-SOD1^SH^ (**a**), and incubated at 37° C with 5 mM of MG for 1 hour (**b**), 5 hours (**c**), 24 hours (**d**), and 48 hours (**e**).


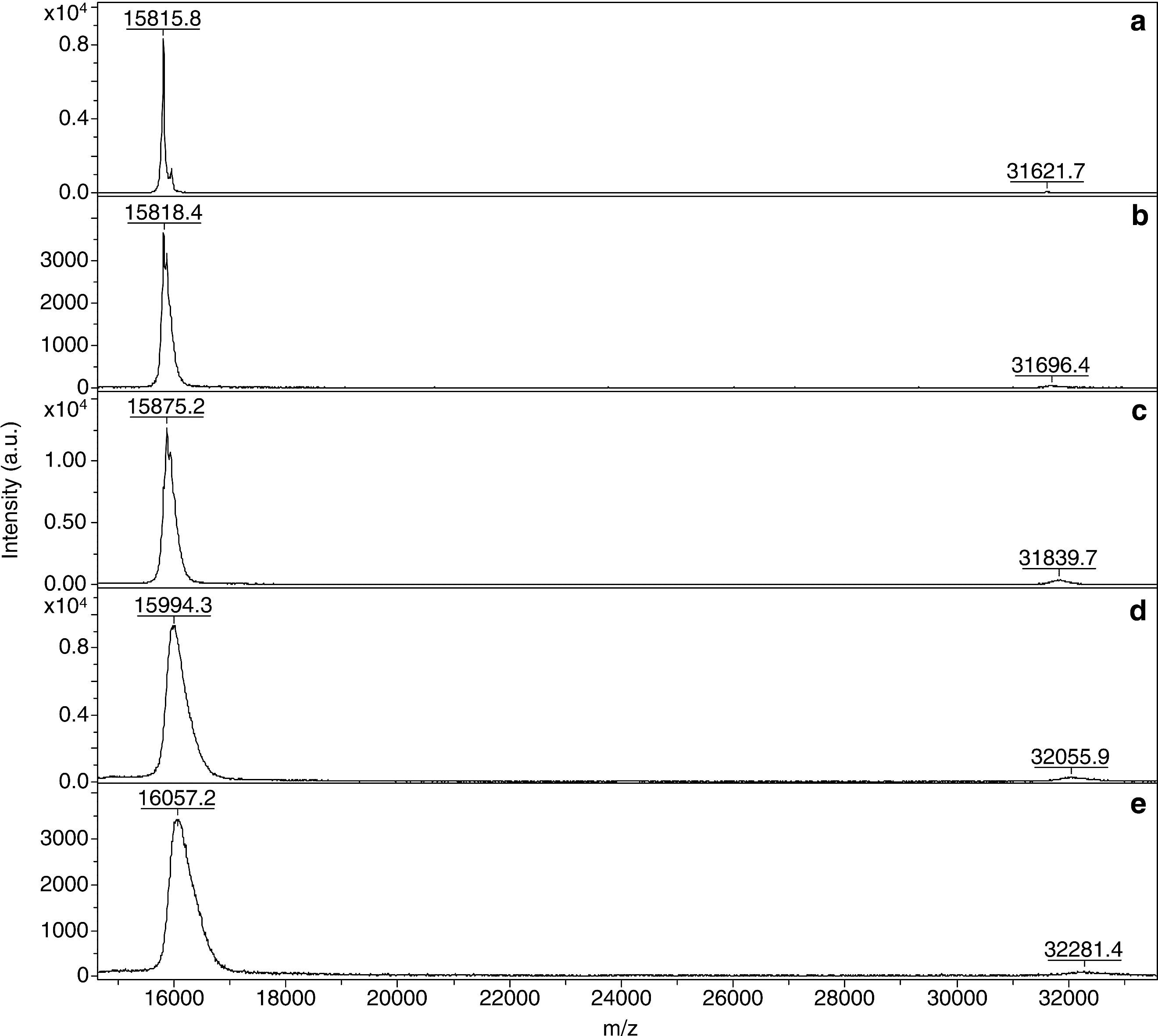


**Figure S2** High *m/z* range MALDI-TOF spectra of control E,Zn-SOD1^SH^ (**a**), and incubated at 37° C with 5 mM of MG for 1 hour (**b**), 5 hours (**c**), 24 hours (**d**), and 48 hours (**e**).


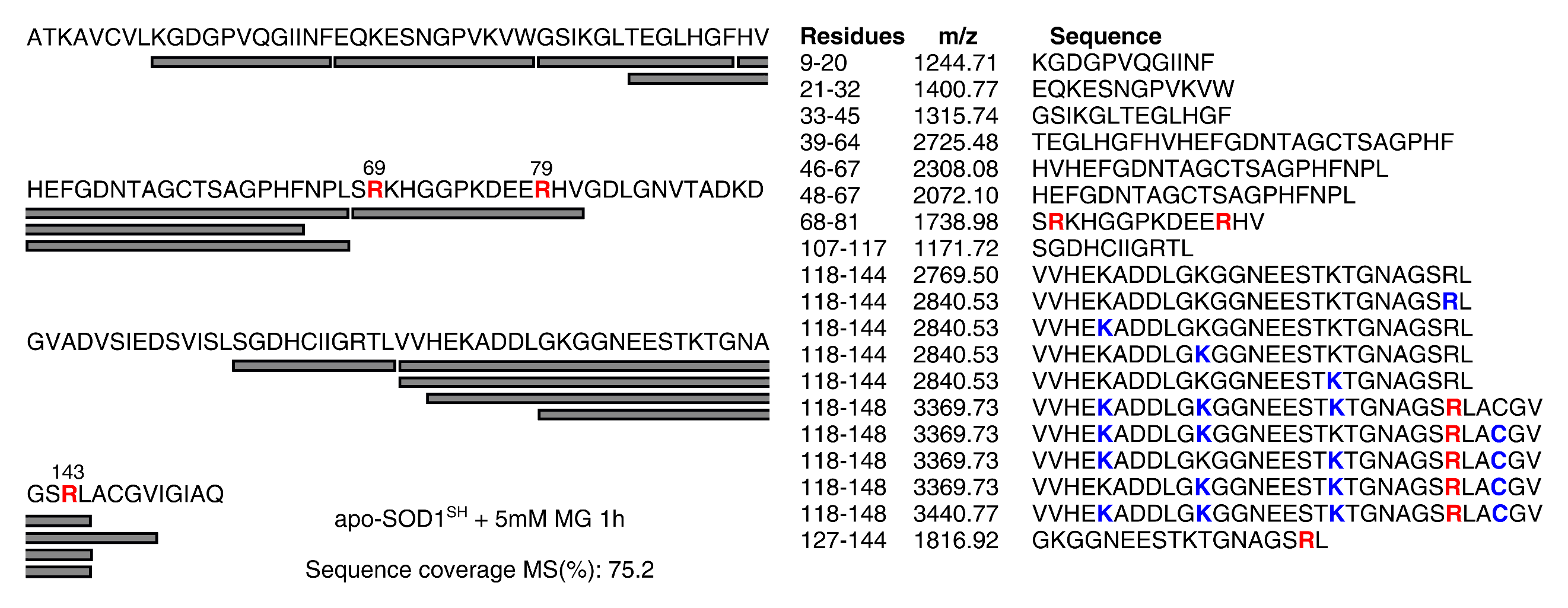


**Figure S3** Fragments of the apo-SOD1^SH^ (incubated with 5 mM of MG for 1 hour) detected by MALDI-TOF upon digestion with chymotrypsin. The residues potentially involved in the reaction with MG, responsible of an increment of 54 or 72 *m/z*, are indicated in red and in blue respectively.


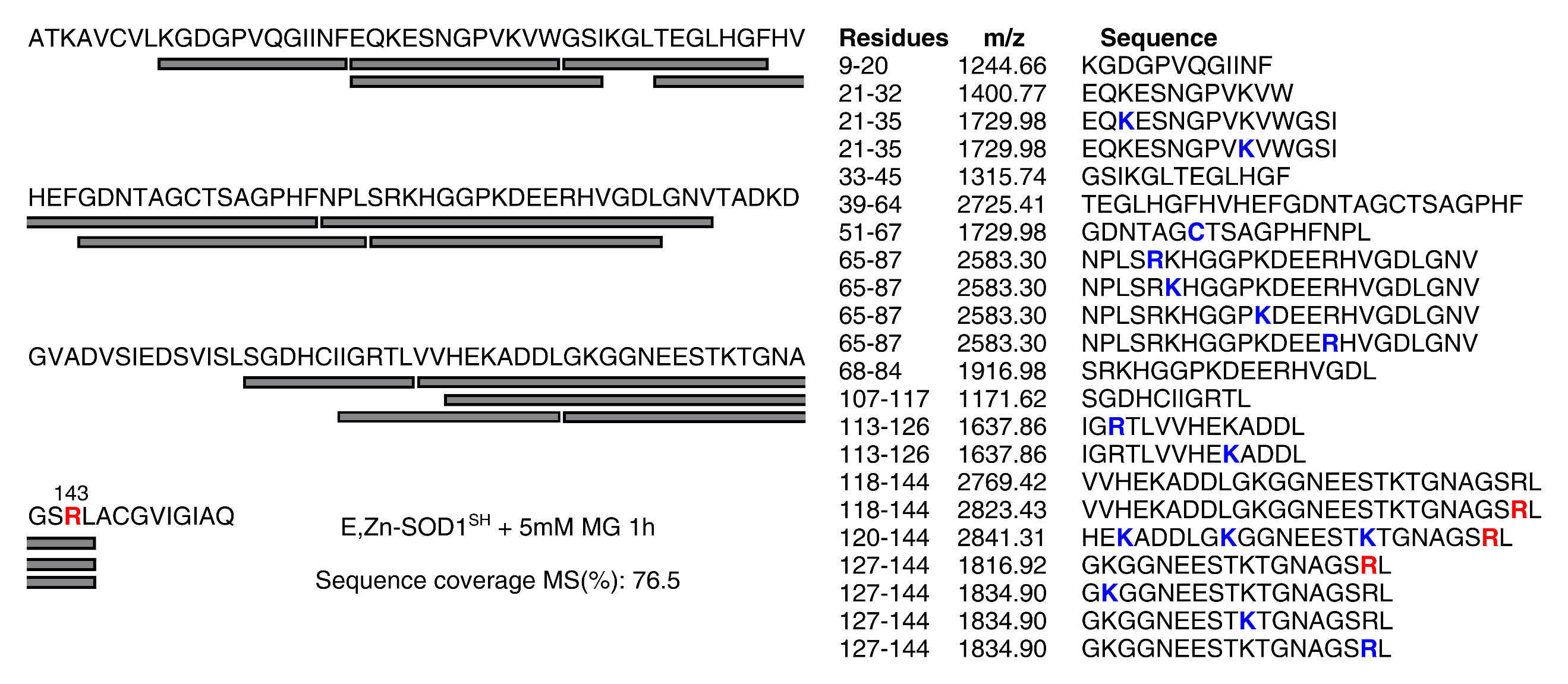


**Figure S4** Fragments of the E,Zn-SOD1^SH^ (incubated with 5 mM of MG for 1 hour) detected by MALDI-TOF upon digestion with chymotrypsin. The residues potentially involved in the reaction with MG, responsible of an increment of 54 and 72 *m/z*, are indicated in red and in blue respectively.





**Figure S5** 2D ^1^H, ^15^N NMR spectra of apo-SOD1^SH^ (left) and E,Zn-SOD1^SH^ (right) at different time points of the reaction with 1 MG. From top to bottom: control (no MG), 1 hour, 6 hours, 12 hours.

**
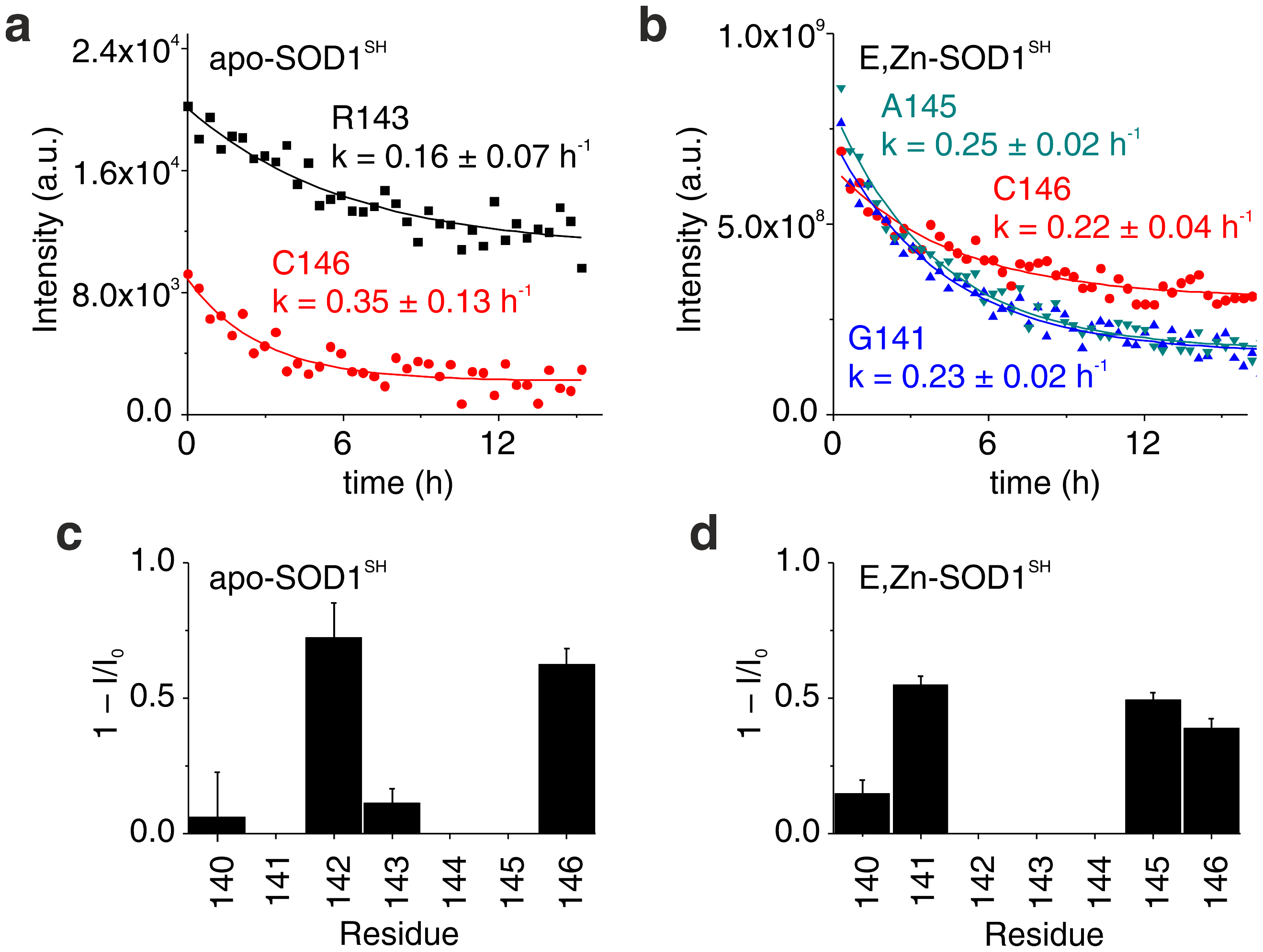
**

**Figure S6.** (**a**,**b**) Plots of signal intensity *vs.* time of residues close to R143 during the reaction of apo-SOD1^SH^ (**a**) and E,Zn-SOD1^SH^ (**b**) with 1 mM MG. Best-fit mono-exponential curves and apparent rate constant values are shown in the corresponding colour. (**c**,**d**) Plots of signal loss for residues close to R143 of apo-SOD1^SH^ (**c**) and E,Zn-SOD1^SH^ (**d**) after 1-hour reaction with 1 mM MG.
